# Supplementary material for: Variability among the Isolates of Broad Bean Mottle Virus and Encapsidation of Host RNAs
Source: Pathogens. 2022 Jul 21;11(7):817. doi: 10.3390/pathogens11070817 (PMC9321246; doi:10.3390/pathogens11070817)
Supplement: Supplementary file 1 [file Figure S4 3a Alignment.pdf › Figure S4 3a Alignment.pdf]

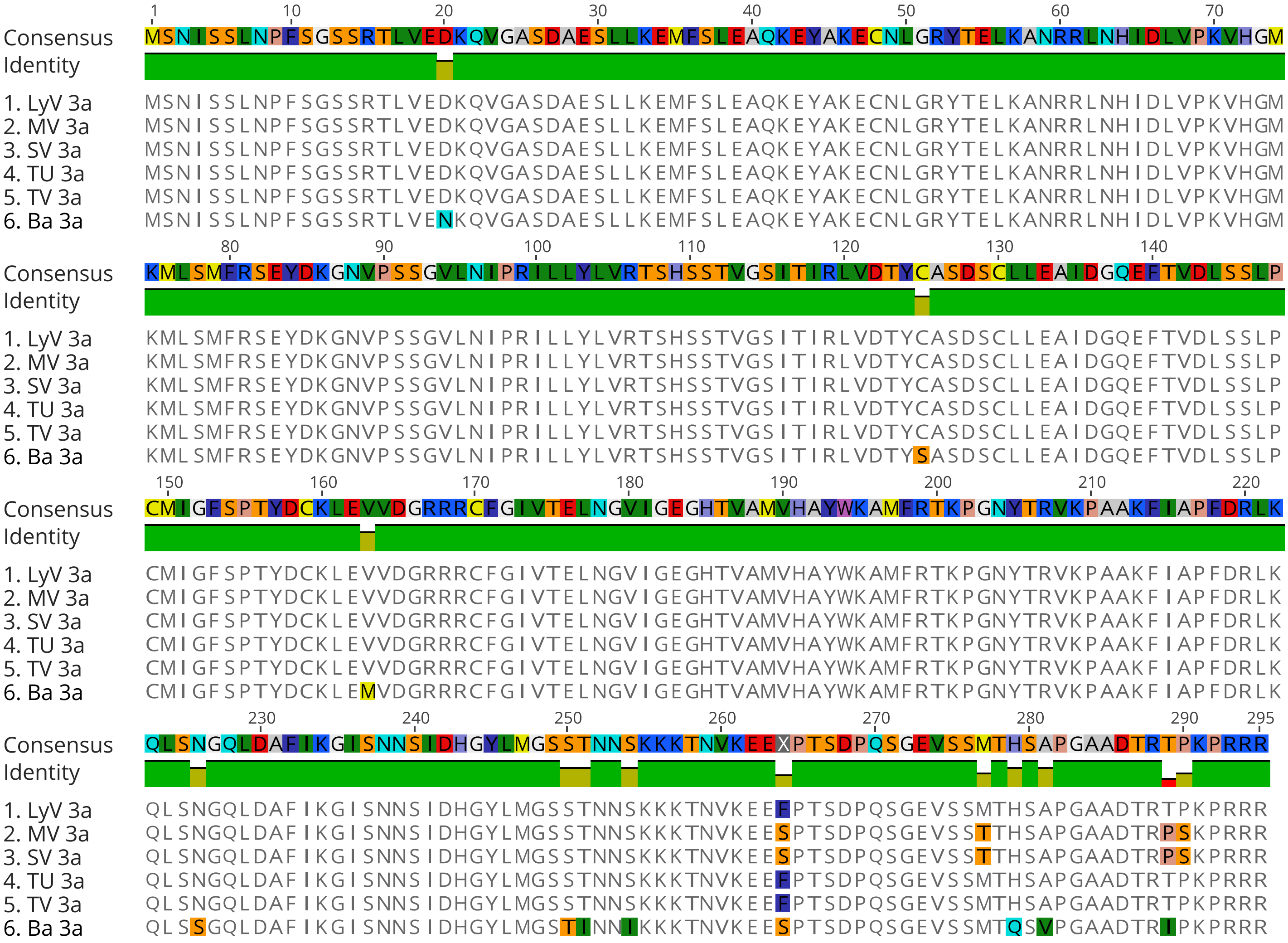
1 10 20 30 40 50 60 70
Consensus MSNISSLNPFSGSSRTLVEDKQVGASDAESLLKEMFSLEAQKEYAKECNLGRYTELKANRRLNHIDLVPKVHGM
Identity
1. LyV 3a MSNISSLNPFSGSSRTLVEDKQVGASDAESLLKEMFSLEAQKEYAKECNLGRYTELKANRRLNHIDLVPKVHGM
2. MV 3a MSNISSLNPFSGSSRTLVEDKQVGASDAESLLKEMFSLEAQKEYAKECNLGRYTELKANRRLNHIDLVPKVHGM
3. SV 3a MSNISSLNPFSGSSRTLVEDKQVGASDAESLLKEMFSLEAQKEYAKECNLGRYTELKANRRLNHIDLVPKVHGM
4. TU 3a MSNISSLNPFSGSSRTLVEDKQVGASDAESLLKEMFSLEAQKEYAKECNLGRYTELKANRRLNHIDLVPKVHGM
5. TV 3a MSNISSLNPFSGSSRTLVEDKQVGASDAESLLKEMFSLEAQKEYAKECNLGRYTELKANRRLNHIDLVPKVHGM
6. Ba 3a MSNISSLNPFSGSSRTLVENKQVGASDAESLLKEMFSLEAQKEYAKECNLGRYTELKANRRLNHIDLVPKVHGM
80 90 100 110 120 130 140
Consensus KMLSMFRSEYDKGNVPSSGVLNIPRILLYLVRTSHSSTVGSITIRLVDTYCASDSCLLEAIDGQEFTVDLSSLP
Identity
1. LyV 3a KMLSMFRSEYDKGNVPSSGVLNIPRILLYLVRTSHSSTVGSITIRLVDTYCASDSCLLEAIDGQEFTVDLSSLP
2. MV 3a KMLSMFRSEYDKGNVPSSGVLNIPRILLYLVRTSHSSTVGSITIRLVDTYCASDSCLLEAIDGQEFTVDLSSLP
3. SV 3a KMLSMFRSEYDKGNVPSSGVLNIPRILLYLVRTSHSSTVGSITIRLVDTYCASDSCLLEAIDGQEFTVDLSSLP
4. TU 3a KMLSMFRSEYDKGNVPSSGVLNIPRILLYLVRTSHSSTVGSITIRLVDTYCASDSCLLEAIDGQEFTVDLSSLP
5. TV 3a KMLSMFRSEYDKGNVPSSGVLNIPRILLYLVRTSHSSTVGSITIRLVDTYCASDSCLLEAIDGQEFTVDLSSLP
6. Ba 3a KMLSMFRSEYDKGNVPSSGVLNIPRILLYLVRTSHSSTVGSITIRLVDTYSASDSCLLEAIDGQEFTVDLSSLP
150 160 170 180 190 200 210 220
Consensus CMIGFSPTYDCKLEVVDGRRRCFGIVTELNGVIGEGHTVAMVHAYWKAMFRTKPGNYTRVKPAAKFIAPFDRLK
Identity
1. LyV 3a CMIGFSPTYDCKLEVVDGRRRCFGIVTELNGVIGEGHTVAMVHAYWKAMFRTKPGNYTRVKPAAKFIAPFDRLK
2. MV 3a CMIGFSPTYDCKLEVVDGRRRCFGIVTELNGVIGEGHTVAMVHAYWKAMFRTKPGNYTRVKPAAKFIAPFDRLK
3. SV 3a CMIGFSPTYDCKLEVVDGRRRCFGIVTELNGVIGEGHTVAMVHAYWKAMFRTKPGNYTRVKPAAKFIAPFDRLK
4. TU 3a CMIGFSPTYDCKLEVVDGRRRCFGIVTELNGVIGEGHTVAMVHAYWKAMFRTKPGNYTRVKPAAKFIAPFDRLK
5. TV 3a CMIGFSPTYDCKLEVVDGRRRCFGIVTELNGVIGEGHTVAMVHAYWKAMFRTKPGNYTRVKPAAKFIAPFDRLK
6. Ba 3a CMIGFSPTYDCKLEMVDGRRRCFGIVTELNGVIGEGHTVAMVHAYWKAMFRTKPGNYTRVKPAAKFIAPFDRLK
230 240 250 260 270 280 290 295
Consensus QLSNGQLDAFIKGISNNSIDHGYLMGSSTNNSKKKTNVKEEXPTSDPQSGEVSSMTHSAPGAADTRTPKPRRR
Identity
1. LyV 3a QLSNGQLDAFIKGISNNSIDHGYLMGSSTNNSKKKTNVKEEFPTSDPQSGEVSSMTHSAPGAADTRTPKPRRR
2. MV 3a QLSNGQLDAFIKGISNNSIDHGYLMGSSTNNSKKKTNVKEESPTSDPQSGEVSSTTHSAPGAADTRPSKPRRR
3. SV 3a QLSNGQLDAFIKGISNNSIDHGYLMGSSTNNSKKKTNVKEESPTSDPQSGEVSSTTHSAPGAADTRPSKPRRR
4. TU 3a QLSNGQLDAFIKGISNNSIDHGYLMGSSTNNSKKKTNVKEEFPTSDPQSGEVSSMTHSAPGAADTRTPKPRRR
5. TV 3a QLSNGQLDAFIKGISNNSIDHGYLMGSSTNNSKKKTNVKEEFPTSDPQSGEVSSMTHSAPGAADTRTPKPRRR
6. Ba 3a QLSSGQLDAFIKGISNNSIDHGYLMGSTINNIKKKTNVKEESPTSDPQSGEVSSMTQSVPGAADTRIPKPRRR
