# Supplementary material for: Variability among the Isolates of Broad Bean Mottle Virus and Encapsidation of Host RNAs
Source: Pathogens. 2022 Jul 21;11(7):817. doi: 10.3390/pathogens11070817 (PMC9321246; doi:10.3390/pathogens11070817)
Supplement: Supplementary file 1 [file Figure S5 4a Alignment.pdf › Figure S5 4a Alignment.pdf]

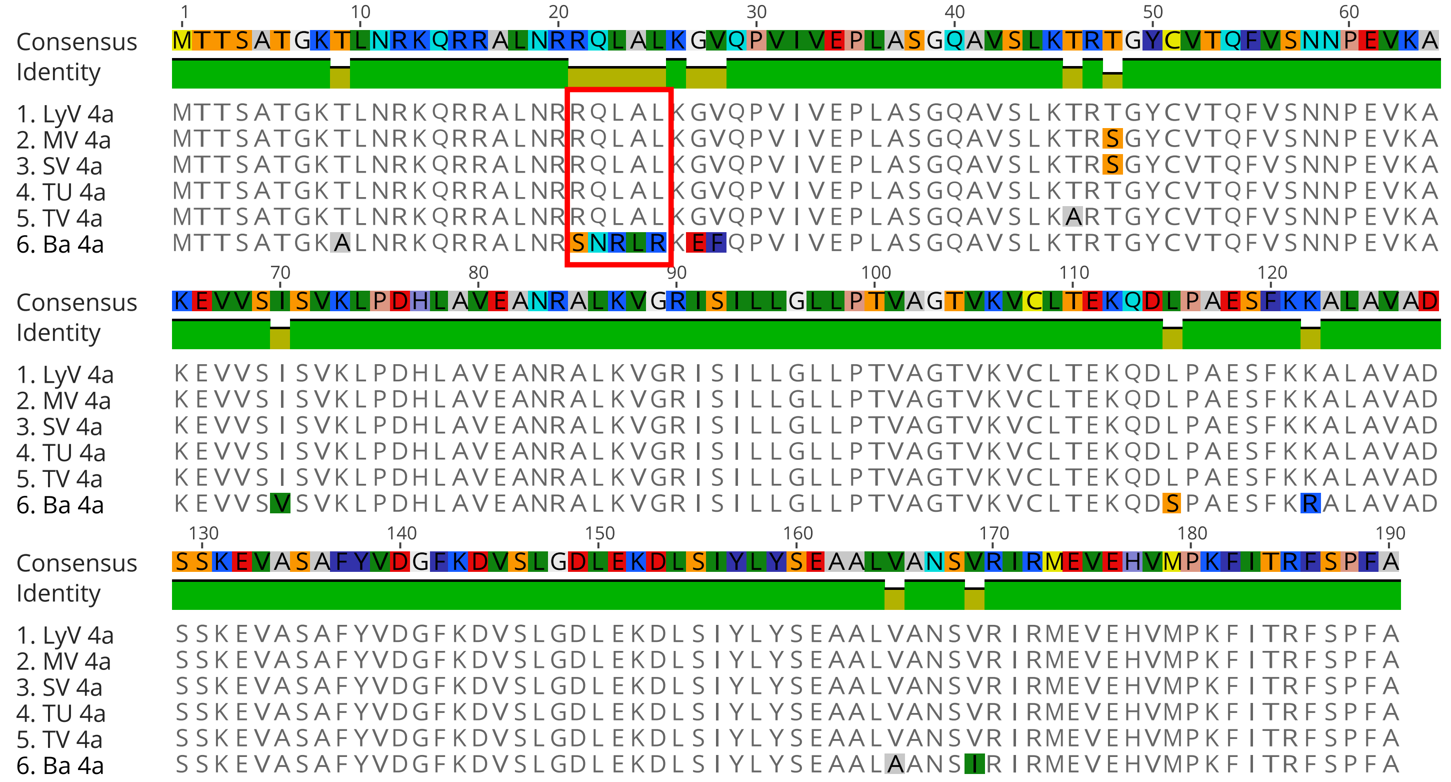
1 10 20 30 40 50 60
Consensus MTTSATGKTLNRKQRRALNRRQLALKGVQPVIVEPLASGQAVSLKTRTGYCVTQFVSNNPEVKA
Identity
1. LyV 4a MTTSATGKTLNRKQRRALNRRQLALKGVQPVIVEPLASGQAVSLKTRTGYCVTQFVSNNPEVKA
2. MV 4a MTTSATGKTLNRKQRRALNRRQLALKGVQPVIVEPLASGQAVSLKTRSGYCVTQFVSNNPEVKA
3. SV 4a MTTSATGKTLNRKQRRALNRRQLALKGVQPVIVEPLASGQAVSLKTRSGYCVTQFVSNNPEVKA
4. TU 4a MTTSATGKTLNRKQRRALNRRQLALKGVQPVIVEPLASGQAVSLKTRTGYCVTQFVSNNPEVKA
5. TV 4a MTTSATGKTLNRKQRRALNRRQLALKGVQPVIVEPLASGQAVSLKARTGYCVTQFVSNNPEVKA
6. Ba 4a MTTSATGKALNRKQRRALNRSNRLRKEFQPVIVEPLASGQAVSLKTRTGYCVTQFVSNNPEVKA
70 80 90 100 110 120
Consensus KEVVSISVKLPDHLAVEANRALKVGRISILLGLLPTVAGTVKVCLTEKQDLPAESFKKALAVAD
Identity
1. LyV 4a KEVVSISVKLPDHLAVEANRALKVGRISILLGLLPTVAGTVKVCLTEKQDLPAESFKKALAVAD
2. MV 4a KEVVSISVKLPDHLAVEANRALKVGRISILLGLLPTVAGTVKVCLTEKQDLPAESFKKALAVAD
3. SV 4a KEVVSISVKLPDHLAVEANRALKVGRISILLGLLPTVAGTVKVCLTEKQDLPAESFKKALAVAD
4. TU 4a KEVVSISVKLPDHLAVEANRALKVGRISILLGLLPTVAGTVKVCLTEKQDLPAESFKKALAVAD
5. TV 4a KEVVSISVKLPDHLAVEANRALKVGRISILLGLLPTVAGTVKVCLTEKQDLPAESFKKALAVAD
6. Ba 4a KEVVSVSVKLPDHLAVEANRALKVGRISILLGLLPTVAGTVKVCLTEKQDSPAESFKRALAVAD
130 140 150 160 170 180 190
Consensus SSKEVASAFYVDGFKDVSLGDLEKDLSIYLYSEAALVANSVRIRMEVEHVMPKFITRFSPFA
Identity
1. LyV 4a SSKEVASAFYVDGFKDVSLGDLEKDLSIYLYSEAALVANSVRIRMEVEHVMPKFITRFSPFA
2. MV 4a SSKEVASAFYVDGFKDVSLGDLEKDLSIYLYSEAALVANSVRIRMEVEHVMPKFITRFSPFA
3. SV 4a SSKEVASAFYVDGFKDVSLGDLEKDLSIYLYSEAALVANSVRIRMEVEHVMPKFITRFSPFA
4. TU 4a SSKEVASAFYVDGFKDVSLGDLEKDLSIYLYSEAALVANSVRIRMEVEHVMPKFITRFSPFA
5. TV 4a SSKEVASAFYVDGFKDVSLGDLEKDLSIYLYSEAALVANSVRIRMEVEHVMPKFITRFSPFA
6. Ba 4a SSKEVASAFYVDGFKDVSLGDLEKDLSIYLYSEAALAANSIRIRMEVEHVMPKFITRFSPFA
