# Supplementary material for: Variability among the Isolates of Broad Bean Mottle Virus and Encapsidation of Host RNAs
Source: Pathogens. 2022 Jul 21;11(7):817. doi: 10.3390/pathogens11070817 (PMC9321246; doi:10.3390/pathogens11070817)
Supplement: Supplementary file 1 [file pathogens-11-00817-s001.zip › Supplementary Materials.pdf]

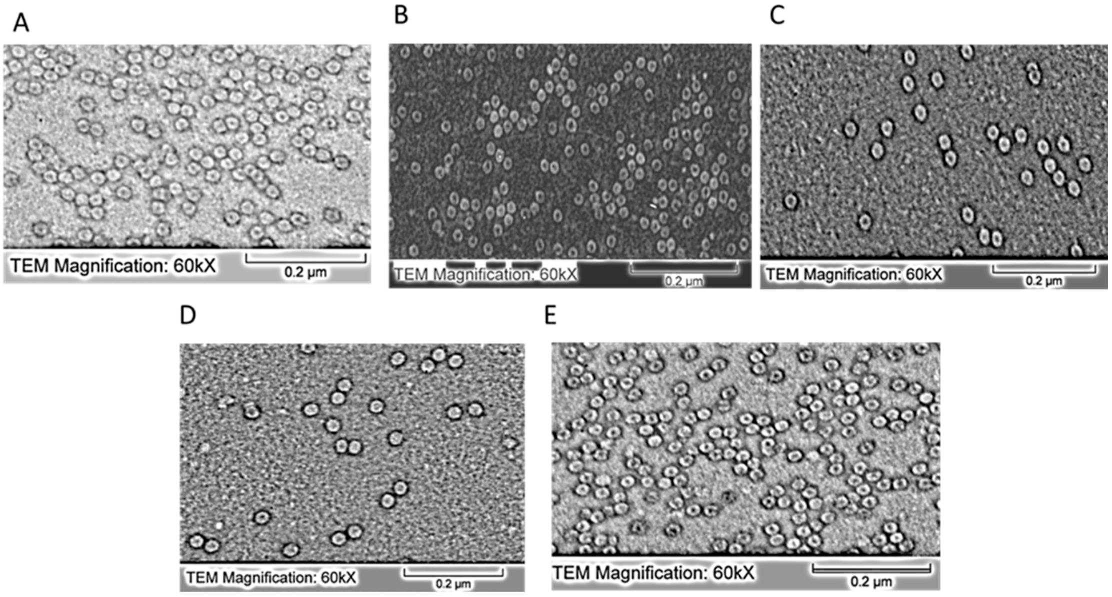

Figure S1. Transmission electron microscopy of extensively purified preparations of five BBMV isolates from the broad bean leaves. A) LyV, B) MV, C) SV, D) TU, E) TV. The characteristic spherical virions with an average diameter of 27-30nm (n=20, scale µm) can be observed.

Foxit Reader PDF Document

Figure S2. The MSA of amino acid sequences of proteins 1a of five new BBMV isolates as compared to the Ba isolate. The identical aa are marked with the green color whereas the polymorphic regions with the olive color. The numerical scale on top marks the positions of amino acids.

Foxit Reader PDF Document

Figure S3. The MSA of amino acid sequences of proteins 2a of five new BBMV isolates as compared to Ba isolate. The identical aa are marked with the green color whereas the polymorphic regions with the olive color. The numerical scale on top marks the positions of amino acids. The conserved GDD motif is highlighted by red box.

Foxit Reader PDF Document

Figure S4. The MSA of amino acid sequences of proteins 3a of five new BBMV isolates as compared to Ba isolate. The identical aa are marked with the green color whereas the polymorphic regions with the olive color. The numerical scale on top marks the positions of amino acids.

Foxit Reader PDF Document

Figure S5. The MSA of amino acid sequences of 4a protein (CP) of five new BBMV isolates as compared to Ba isolate. The identical aa are marked with the green color whereas the polymorphic regions with the olive color. The numerical scale on top marks the positions of amino acids. The N-terminal RQLAL and SNLRL motifs are highlighted by the red box.

Table S1. Summary of distribution of the RNA segments encapsidated in the BBMV isolates [1].

Strains	LyV		MV		SV		TU		TV	
	# of Reads	% of Reads	# of Reads	% of Reads	# of Reads	% of Reads	# of Reads	% of Reads	# of Reads	% of Reads
Total Viral	69229051		68221151		68160355		71589085		77025168	
RNA1	32299471	47.93%	28728260	43.51%	35852206	54.04%	34667152	49.81%	27797945	37.53%
RNA2	26041595	38.65%	25536067	38.68%	23166369	34.92%	26628222	38.26%	33745377	45.56%
RNA3	9044034	13.42%	11761271	17.81%	7330627	11.05%	8306709	11.93%	12518558	16.90%
Total Viral	**67385100**		**66025598**		**66349202**		**69602083**		**74061880**	
Non-viral	1843951	2.66%	2195553	3.22%	1811153	2.66%	1987002	2.78%	2963288	3.85%

[1] Based on the number of reads of the NGS RNA Seq.

Table S2. Summary of reads that mapped to BBMV and Broad bean references.

	LyV		MV		SV		TU		TV	
	Reads	% of total	Reads	% of total	Reads	% of total	Reads	% of total	Reads	% of total
Total	69229051		68221151		68160354		71589083		77025168	
RNA1	32649320	47.16	29071640	42.61	36218467	53.14	35025952	48.93	28138605	36.53
RNA2	26305408	38.00	25822064	37.85	23415810	34.35	26909577	37.59	34146453	44.33
RNA3	9101173	13.15	12052263	17.67	7457385	10.94	8449249	11.80	12761912	16.57
Viral	**68055901**	**98.31**	**66945967**	**98.13**	**67091662**	98.43	**70384778**	98.32	**75046970**	97.43
Non-Viral	1173150	1.69	1275184	1.87	1068692	1.57	1204305	1.68	1978198	2.57
Host	789386	1.14	913243	1.34	759606	1.11	934017	1.30	1501568	1.95
Unassigned	383764		361941		309086		270288		476630	

Table S3. Summary of reads mapped to host RNAs encoded in different cellular compartments.

	**LyV**		**MV**		**SV**		**TU**		**TV**	
	Reads	% Of host reads	Reads	% Of host reads	Reads	% Of host reads	Reads	% Of host reads	Reads	% Of host reads
**Nuclear**	**788848**	**99.93**	**911790**	**99.84**	**758696**	**99.88**	**932412**	**99.83**	**1500263**	**99.91**
mRNA	372824	47.23	391850	42.91	293364	38.62	304523	32.60	434434	28.93
rRNA	383611	48.60	498545	54.59	443941	58.44	608020	65.10	1030021	68.60
Others	32413	4.11	21395	2.34	21391	2.82	19869	2.13	35808	2.38
**Mitochondrial**	**2**	**0.00**	**46**	**0.01**	**7**	**0.00**	**16**	**0.00**	**13**	**0.00**
mRNA	0	0.00	18	0.00	4	0.00	6	0.00	4	0.00
rRNA	2	0.00	28	0.00	3	0.00	10	0.00	8	0.00
Others	0	0.00	0	0.00	0	0.00	0	0.00	1	0.00
**Plastid**	**536**	**0.07**	**1407**	**0.15**	**903**	**0.12**	**1589**	**0.17**	**1292**	**0.09**
mRNA	129	0.02	280	0.03	62	0.01	545	0.06	313	0.02
rRNA	366	0.05	1082	0.12	817	0.11	990	0.11	907	0.06
Others	41	0.01	45	0.00	24	0.00	54	0.01	72	0.00
**Total**	**789386**		**913243**		**759606**		**934017**		**1501568**	
TE	39627		26608		15880		20667		32310	

Table S4. The major host mRNAs encapsidated in five BBMV isolates.

Reference	Transcript Name	Number of Reads				
		LyV	MV	SV	TU	TV
v.faba_CSFL_reftransV2_0011664	photosystem II repair protein PSB27-H1, chloroplastic OS=Cicer arietinum GN=LOC101500783 PE=3 SV=1	171484	176866	157899	198165	222801
v.faba_csfl_reftransV1_0044601	NA	62872	28410	28765	23224	50004
v.faba_csfl_reftransV1_0015684	40S ribosomal protein S25 (Fragment) OS=Cricetulus griseus GN=I79_020113 PE=4 SV=1	35890	21939	12369	14823	22730
v.faba_CSFL_reftransV2_0001947	Uncharacterized protein OS=Lupinus angustifolius GN=TanjilG_03599 PE=4 SV=1	1507	1205	912	1312	1513
v.faba_csfl_reftransV1_0056153	Fructose-bisphosphate aldolase OS=Medicago truncatula GN=MTR_4g071860 PE=3 SV=1	850	805	573	833	1145
v.faba_csfl_reftransV1_0012340	Uncharacterized protein OS=Glycine max PE=4 SV=1	782	1018	661	1084	1407
v.faba_CSFL_reftransV2_0006213	heat shock cognate 70 kDa protein 2-like OS=Cicer arietinum GN=LOC101502845 PE=3 SV=1	716	315	281	416	468
v.faba_CSFL_reftransV2_0000502	Uncharacterized protein OS=Medicago truncatula GN=MTR_0052s0170 PE=4 SV=1	704	562	399	552	1097
v.faba_csfl_reftransV1_0037233	Uncharacterized protein OS=Glycine max PE=4 SV=1	699	944	652	1042	1255
v.faba_CSFL_reftransV2_0009840	Uncharacterized protein OS=Lupinus angustifolius GN=TanjilG_03599 PE=4 SV=1	673	967	663	1014	1234
v.faba_csfl_reftransV1_0033771	Ribosome biogenesis GTP-binding protein YsxC OS=Medicago truncatula GN=MTR_3g109540 PE=3 SV=1	160	50596	27161	28	52
v.faba_CSFL_reftransV2_0032752	GTP-binding protein At2g22870 OS=Cicer arietinum GN=LOC101508363 PE=3 SV=1	132	50117	27189	25	41
v.faba_csfl_reftransV1_0043170	Uncharacterized protein OS=Glycine max PE=4 SV=1	529	1504	765	1215	835
v.faba_csfl_reftransV1_0011896	Uncharacterized protein OS=Medicago truncatula GN=MTR_5g089380 PE=4 SV=1	575	1202	572	692	470
v.faba_CSFL_reftransV2_0019991	Transmembrane protein, putative OS=Medicago truncatula GN=MTR_5g037260 PE=4 SV=1	369	970	488	498	393
v.faba_csfl_reftransV1_0037237	Uncharacterized protein OS=Glycine max PE=4 SV=1	441	568	378	515	1387
v.faba_csfl_reftransV1_0037958	Uncharacterized protein OS=Medicago truncatula GN=MTR_8g073305 PE=4 SV=1	258	36	23	214	1151

1) The reads that did not map to BBMV reference were mapped to the transcriptome database of *Vicia faba*. Then the top ten most mapped reads to the mRNA transcript for the five BBMV isolates were extracted. The common transcripts were combined such that all five strains have ten of them as top hits. The reads for organellar RNAs were very low in BBMV and thus are not included in this table.

Table S5. Summary of the transposable elements among the encapsidated host mRNA transcripts[1)]

Transposable Elements		Number of Reads				
**Class I**	Order	LyV	MV	SV	TU	TV
	DIRS	374	333	343	482	651
	LARD	13	15	13	19	43
	LINE	33289	21145	11784	14247	21912
	LTR-Copia	3179	2646	2193	3038	4927
	LTR-Gypsy	170	147	65	89	148
	LTR SINE	100	266	99	130	164
	TRIM	3	22	10	19	11
**Class II**	Unknown	37	36	23	23	41
	Helitron	24	19	16	22	46
	MITE	0	3	5	1	2
	TIR	308	136	59	112	312
**Unknown**		**2130**	**1840**	**1270**	**2485**	**4053**
**Total**		**39627**	**26608**	**15880**	**20667**	**32310**

[1)] The table shows the portion of host transcripts that correspond to transposable elements. The reads that mapped to the mRNA transcripts were then mapped to the TE database that was downloaded from RepetDB (Amselem et al., 2019). Retro transposons (Class I) like LINE and LTR predominate among the reads shown in this table.

Table S6. The comparison of the average number of reads mapped to host RNAs originating from three different host cellular compartments in BMV vs BBMV [1)]

Compartments	RNA	Average Reads Mapped	
		**BMV**	**BBMV**
Nuclear	mRNA	23172	359399
Nuclear	rRNA	976	592827.6
Nuclear	genomic	1708.5	26175.2
Mitochondrial	mRNA	6713	6.4
Mitochondrial	rRNA	552	10.2
Plastid	mRNA	16958.5	265.8
Plastid	rRNA	370	832.4
Total		**50450**	**979516.6**

[1)] For BMV, the reads mapped to host RNAs were averaged between two different virus purifications from barley (Shrestha et al., 2018) whereas for BBMV, the data were averaged from five virion purifications. The average number of reads for BBMV that mapped to the host RNAs were 19.4 times higher than that for BMV. Among the BMV host reads, little over 50% of reads mapped to the host nuclear RNAs, whereas for BBMV, more than 99% reads mapped to the host nuclear RNAs and <0.1% reads mapped to organellar RNAs.
