# Supplementary material for: Variability among the Isolates of Broad Bean Mottle Virus and Encapsidation of Host RNAs
Source: Pathogens. 2022 Jul 21;11(7):817. doi: 10.3390/pathogens11070817 (PMC9321246; doi:10.3390/pathogens11070817)
Supplement: Supplementary file 1 [file pathogens-11-00817-s001.zip › Figure S2 1a Alignment.pdf]

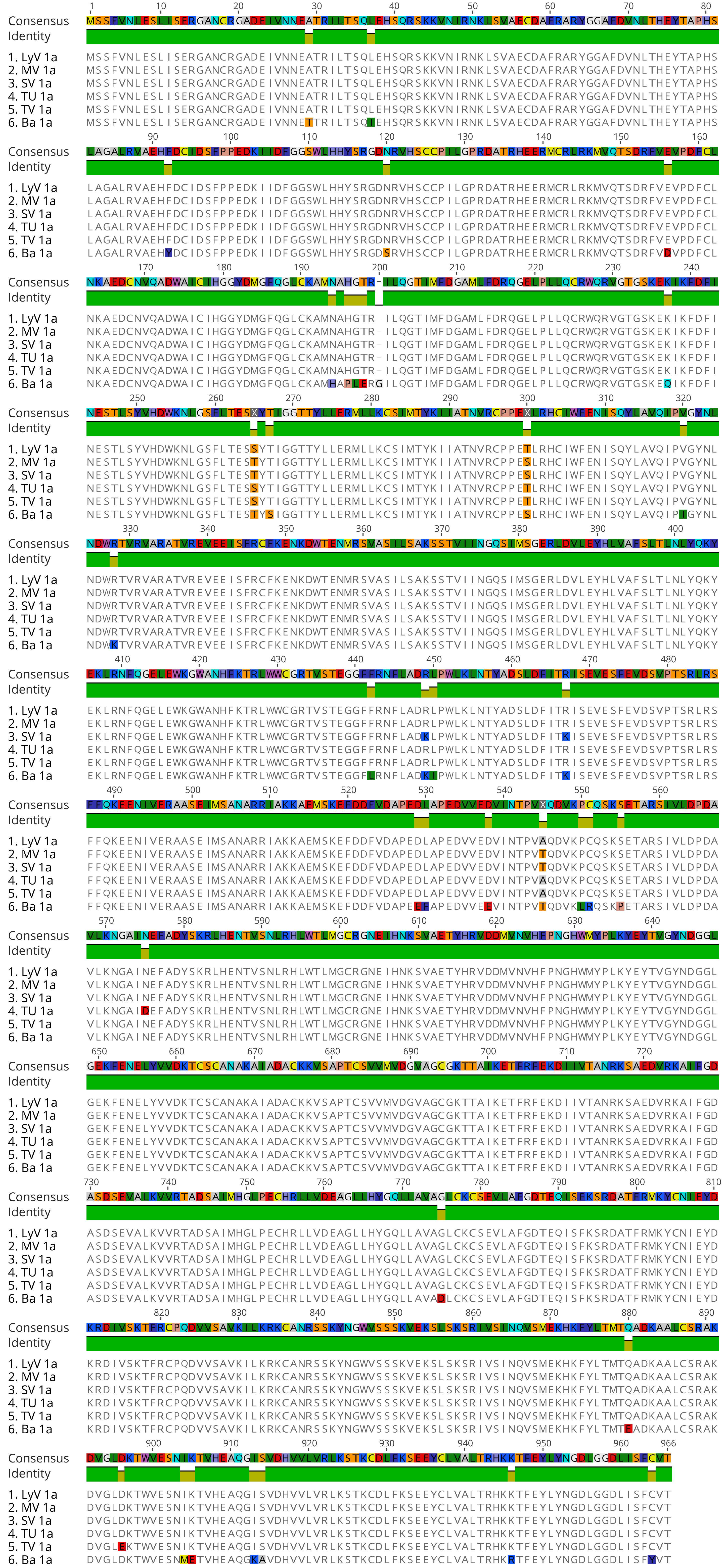
1 10 20 30 40 50 60 70 80
Consensus MSSFVNLESLISERGANCRGADEIVNNEATRILTSQLEHSQRSKKVNIRNKLSVAECDAFRARYGGAFDVNLTHEYTAPHS
Identity
1. LyV 1a MSSFVNLESLISERGANCRGADEIVNNEATRILTSQLEHSQRSKKVNIRNKLSVAECDAFRARYGGAFDVNLTHEYTAPHS
2. MV 1a MSSFVNLESLISERGANCRGADEIVNNEATRILTSQLEHSQRSKKVNIRNKLSVAECDAFRARYGGAFDVNLTHEYTAPHS
3. SV 1a MSSFVNLESLISERGANCRGADEIVNNEATRILTSQLEHSQRSKKVNIRNKLSVAECDAFRARYGGAFDVNLTHEYTAPHS
4. TU 1a MSSFVNLESLISERGANCRGADEIVNNEATRILTSQLEHSQRSKKVNIRNKLSVAECDAFRARYGGAFDVNLTHEYTAPHS
5. TV 1a MSSFVNLESLISERGANCRGADEIVNNEATRILTSQLEHSQRSKKVNIRNKLSVAECDAFRARYGGAFDVNLTHEYTAPHS
6. Ba 1a MSSFVNLESLISERGANCRGADEIVNNETTRILTSQIEHSQRSKKVNIRNKLSVAECDAFRARYGGAFDVNLTHEYTAPHS
90 100 110 120 130 140 150 160
Consensus LAGALRVAEHFDCIDSFPPEDKIIDFGGSWLHHYSRGDNRVHSCCPILGPRDATRHEERMCRLRKMVQTSDRFVEVPDFCL
Identity
1. LyV 1a LAGALRVAEHFDCIDSFPPEDKIIDFGGSWLHHYSRGDNRVHSCCPILGPRDATRHEERMCRLRKMVQTSDRFVEVPDFCL
2. MV 1a LAGALRVAEHFDCIDSFPPEDKIIDFGGSWLHHYSRGDNRVHSCCPILGPRDATRHEERMCRLRKMVQTSDRFVEVPDFCL
3. SV 1a LAGALRVAEHFDCIDSFPPEDKIIDFGGSWLHHYSRGDNRVHSCCPILGPRDATRHEERMCRLRKMVQTSDRFVEVPDFCL
4. TU 1a LAGALRVAEHFDCIDSFPPEDKIIDFGGSWLHHYSRGDNRVHSCCPILGPRDATRHEERMCRLRKMVQTSDRFVEVPDFCL
5. TV 1a LAGALRVAEHFDCIDSFPPEDKIIDFGGSWLHHYSRGDNRVHSCCPILGPRDATRHEERMCRLRKMVQTSDRFVEVPDFCL
6. Ba 1a LAGALRVAEHYDCIDSFPPEDKIIDFGGSWLHHYSRGDSRVHSCCPILGPRDATRHEERMCRLRKMVQTSDRFVDVPDFCL
170 180 190 200 210 220 230 240
Consensus NKAEDCNVQADWAICIHGGYDMGFQGLCKAMNAHGTR-ILQGTIMFDGAMLFDRQGELPLLQCRWQRVGTGSKEKIKFDFI
Identity
1. LyV 1a NKAEDCNVQADWAICIHGGYDMGFQGLCKAMNAHGTR-ILQGTIMFDGAMLFDRQGELPLLQCRWQRVGTGSKEKIKFDFI
2. MV 1a NKAEDCNVQADWAICIHGGYDMGFQGLCKAMNAHGTR-ILQGTIMFDGAMLFDRQGELPLLQCRWQRVGTGSKEKIKFDFI
3. SV 1a NKAEDCNVQADWAICIHGGYDMGFQGLCKAMNAHGTR-ILQGTIMFDGAMLFDRQGELPLLQCRWQRVGTGSKEKIKFDFI
4. TU 1a NKAEDCNVQADWAICIHGGYDMGFQGLCKAMNAHGTR-ILQGTIMFDGAMLFDRQGELPLLQCRWQRVGTGSKEKIKFDFI
5. TV 1a NKAEDCNVQADWAICIHGGYDMGFQGLCKAMNAHGTR-ILQGTIMFDGAMLFDRQGELPLLQCRWQRVGTGSKEKIKFDFI
6. Ba 1a NKAEDCNVQADWAICIHGGYDMGFQGLCKAMHAPLERGILQGTIMFDGAMLFDRQGELPLLQCRWQRVGTGSKEQIKFDFI
250 260 270 280 290 300 310 320
Consensus NESTLSYVHDWKNLGSFLTESXYTIGGTTYLLERMLLKCSIMTYKIIATNVRCPPEXLRHCIWFENISQYLAVQIPVGYNL
Identity
1. LyV 1a NESTLSYVHDWKNLGSFLTESSYTIGGTTYLLERMLLKCSIMTYKIIATNVRCPPETLRHCIWFENISQYLAVQIPVGYNL
2. MV 1a NESTLSYVHDWKNLGSFLTESTYTIGGTTYLLERMLLKCSIMTYKIIATNVRCPPESLRHCIWFENISQYLAVQIPVGYNL
3. SV 1a NESTLSYVHDWKNLGSFLTESTYTIGGTTYLLERMLLKCSIMTYKIIATNVRCPPESLRHCIWFENISQYLAVQIPVGYNL
4. TU 1a NESTLSYVHDWKNLGSFLTESSYTIGGTTYLLERMLLKCSIMTYKIIATNVRCPPETLRHCIWFENISQYLAVQIPVGYNL
5. TV 1a NESTLSYVHDWKNLGSFLTESSYTIGGTTYLLERMLLKCSIMTYKIIATNVRCPPETLRHCIWFENISQYLAVQIPVGYNL
6. Ba 1a NESTLSYVHDWKNLGSFLTESTYSIGGTTYLLERMLLKCSIMTYKIIATNVRCPPESLRHCIWFENISQYLAVQIPIGYNL
330 340 350 360 370 380 390 400
Consensus NDWRTVRVARATVREVEEISFRCFKENKDWTENMRSVASILSAKSSTVIINGQSIMSGERLDVLEYHLVAFSLTLNLYQKY
Identity
1. LyV 1a NDWRTVRVARATVREVEEISFRCFKENKDWTENMRSVASILSAKSSTVIINGQSIMSGERLDVLEYHLVAFSLTLNLYQKY
2. MV 1a NDWRTVRVARATVREVEEISFRCFKENKDWTENMRSVASILSAKSSTVIINGQSIMSGERLDVLEYHLVAFSLTLNLYQKY
3. SV 1a NDWRTVRVARATVREVEEISFRCFKENKDWTENMRSVASILSAKSSTVIINGQSIMSGERLDVLEYHLVAFSLTLNLYQKY
4. TU 1a NDWRTVRVARATVREVEEISFRCFKENKDWTENMRSVASILSAKSSTVIINGQSIMSGERLDVLEYHLVAFSLTLNLYQKY
5. TV 1a NDWRTVRVARATVREVEEISFRCFKENKDWTENMRSVASILSAKSSTVIINGQSIMSGERLDVLEYHLVAFSLTLNLYQKY
6. Ba 1a NDWKTVRVARATVREVEEISFRCFKENKDWTENMRSVASILSAKSSTVIINGQSIMSGERLDVLEYHLVAFSLTLNLYQKY
410 420 430 440 450 460 470 480
Consensus EKLRNFQGELEWKGWANHFKTRLWWCGRTVSTEGGFFRNFLADRLPWLKLNTYADSLDFITRISEVESFEVDSVPTSRLRS
Identity
1. LyV 1a EKLRNFQGELEWKGWANHFKTRLWWCGRTVSTEGGFFRNFLADRLPWLKLNTYADSLDFITRISEVESFEVDSVPTSRLRS
2. MV 1a EKLRNFQGELEWKGWANHFKTRLWWCGRTVSTEGGFFRNFLADRLPWLKLNTYADSLDFITRISEVESFEVDSVPTSRLRS
3. SV 1a EKLRNFQGELEWKGWANHFKTRLWWCGRTVSTEGGFFRNFLADKLPWLKLNTYADSLDFITKISEVESFEVDSVPTSRLRS
4. TU 1a EKLRNFQGELEWKGWANHFKTRLWWCGRTVSTEGGFFRNFLADRLPWLKLNTYADSLDFITRISEVESFEVDSVPTSRLRS
5. TV 1a EKLRNFQGELEWKGWANHFKTRLWWCGRTVSTEGGFFRNFLADRLPWLKLNTYADSLDFITRISEVESFEVDSVPTSRLRS
6. Ba 1a EKLRNFQGELEWKGWANHFKTRLWWCGRTVSTEGGFLRNFLADKIPWLKLNTYADSLDFITKISEVESFEVDSVPTSRLRS
490 500 510 520 530 540 550 560
Consensus FFQKEENIVERAASEIMSANARRIAKKAEMSKEFDDFVDAPEDLAPEDVVEDVINTPVXQDVKPCQSKSETARSIVLDPDA
Identity
1. LyV 1a FFQKEENIVERAASEIMSANARRIAKKAEMSKEFDDFVDAPEDLAPEDVVEDVINTPVAQDVKPCQSKSETARSIVLDPDA
2. MV 1a FFQKEENIVERAASEIMSANARRIAKKAEMSKEFDDFVDAPEDLAPEDVVEDVINTPVTQDVKPCQSKSETARSIVLDPDA
3. SV 1a FFQKEENIVERAASEIMSANARRIAKKAEMSKEFDDFVDAPEDLAPEDVVEDVINTPVTQDVKPCQSKSETARSIVLDPDA
4. TU 1a FFQKEENIVERAASEIMSANARRIAKKAEMSKEFDDFVDAPEDLAPEDVVEDVINTPVAQDVKPCQSKSETARSIVLDPDA
5. TV 1a FFQKEENIVERAASEIMSANARRIAKKAEMSKEFDDFVDAPEDLAPEDVVEDVINTPVAQDVKPCQSKSETARSIVLDPDA
6. Ba 1a FFQKEENIVERAASEIMSANARRIAKKAEMSKEFDDFVDAPEEFAPEDVVEEVINTPVTQDVKLRQSKPETARSIVLDPDA
570 580 590 600 610 620 630 640
Consensus VLKNGAINEFADYSKRLHENTVSNLRHLWTLMGCRGNEIHNKSVAETYHRVDDMVNVHFPNGHWMYPLKYEYTVGYNDGGL
Identity
1. LyV 1a VLKNGAINEFADYSKRLHENTVSNLRHLWTLMGCRGNEIHNKSVAETYHRVDDMVNVHFPNGHWMYPLKYEYTVGYNDGGL
2. MV 1a VLKNGAINEFADYSKRLHENTVSNLRHLWTLMGCRGNEIHNKSVAETYHRVDDMVNVHFPNGHWMYPLKYEYTVGYNDGGL
3. SV 1a VLKNGAINEFADYSKRLHENTVSNLRHLWTLMGCRGNEIHNKSVAETYHRVDDMVNVHFPNGHWMYPLKYEYTVGYNDGGL
4. TU 1a VLKNGAIDEFADYSKRLHENTVSNLRHLWTLMGCRGNEIHNKSVAETYHRVDDMVNVHFPNGHWMYPLKYEYTVGYNDGGL
5. TV 1a VLKNGAINEFADYSKRLHENTVSNLRHLWTLMGCRGNEIHNKSVAETYHRVDDMVNVHFPNGHWMYPLKYEYTVGYNDGGL
6. Ba 1a VLKNGAINEFADYSKRLHENTVSNLRHLWTLMGCRGNEIHNKSVAETYHRVDDMVNVHFPNGHWMYPLKYEYTVGYNDGGL
650 660 670 680 690 700 710 720
Consensus GEKFENELYVVDKTCSCANAKAIADACKKVSAPTCSVVMVDGVAGCGKTTAIKETFRFEKDIIVTANRKSAEDVRKAIFGD
Identity
1. LyV 1a GEKFENELYVVDKTCSCANAKAIADACKKVSAPTCSVVMVDGVAGCGKTTAIKETFRFEKDIIVTANRKSAEDVRKAIFGD
2. MV 1a GEKFENELYVVDKTCSCANAKAIADACKKVSAPTCSVVMVDGVAGCGKTTAIKETFRFEKDIIVTANRKSAEDVRKAIFGD
3. SV 1a GEKFENELYVVDKTCSCANAKAIADACKKVSAPTCSVVMVDGVAGCGKTTAIKETFRFEKDIIVTANRKSAEDVRKAIFGD
4. TU 1a GEKFENELYVVDKTCSCANAKAIADACKKVSAPTCSVVMVDGVAGCGKTTAIKETFRFEKDIIVTANRKSAEDVRKAIFGD
5. TV 1a GEKFENELYVVDKTCSCANAKAIADACKKVSAPTCSVVMVDGVAGCGKTTAIKETFRFEKDIIVTANRKSAEDVRKAIFGD
6. Ba 1a GEKFENELYVVDKTCSCANAKAIADACKKVSAPTCSVVMVDGVAGCGKTTAIKETFRFEKDIIVTANRKSAEDVRKAIFGD
730 740 750 760 770 780 790 800 810
Consensus ASDSEVALKVVRTADSAIMHGLPECHRLLVDEAGLLHYGQLLAVAGLCKCSEVLAFGDTEQISFKSRDATFRMKYCNIEYD
Identity
1. LyV 1a ASDSEVALKVVRTADSAIMHGLPECHRLLVDEAGLLHYGQLLAVAGLCKCSEVLAFGDTEQISFKSRDATFRMKYCNIEYD
2. MV 1a ASDSEVALKVVRTADSAIMHGLPECHRLLVDEAGLLHYGQLLAVAGLCKCSEVLAFGDTEQISFKSRDATFRMKYCNIEYD
3. SV 1a ASDSEVALKVVRTADSAIMHGLPECHRLLVDEAGLLHYGQLLAVAGLCKCSEVLAFGDTEQISFKSRDATFRMKYCNIEYD
4. TU 1a ASDSEVALKVVRTADSAIMHGLPECHRLLVDEAGLLHYGQLLAVAGLCKCSEVLAFGDTEQISFKSRDATFRMKYCNIEYD
5. TV 1a ASDSEVALKVVRTADSAIMHGLPECHRLLVDEAGLLHYGQLLAVAGLCKCSEVLAFGDTEQISFKSRDATFRMKYCNIEYD
6. Ba 1a ASDSEVALKVVRTADSAIMHGLPECHRLLVDEAGLLHYGQLLAVADLCKCSEVLAFGDTEQISFKSRDATFRMKYCNIEYD
820 830 840 850 860 870 880 890
Consensus KRDIVSKTFRCPQDVVSAVKILKRKCANRSSKYNGWVSSSKVEKSLSKSRIVSINQVSMEKHKFYLTMTQADKAALCSRAK
Identity
1. LyV 1a KRDIVSKTFRCPQDVVSAVKILKRKCANRSSKYNGWVSSSKVEKSLSKSRIVSINQVSMEKHKFYLTMTQADKAALCSRAK
2. MV 1a KRDIVSKTFRCPQDVVSAVKILKRKCANRSSKYNGWVSSSKVEKSLSKSRIVSINQVSMEKHKFYLTMTQADKAALCSRAK
3. SV 1a KRDIVSKTFRCPQDVVSAVKILKRKCANRSSKYNGWVSSSKVEKSLSKSRIVSINQVSMEKHKFYLTMTQADKAALCSRAK
4. TU 1a KRDIVSKTFRCPQDVVSAVKILKRKCANRSSKYNGWVSSSKVEKSLSKSRIVSINQVSMEKHKFYLTMTQADKAALCSRAK
5. TV 1a KRDIVSKTFRCPQDVVSAVKILKRKCANRSSKYNGWVSSSKVEKSLSKSRIVSINQVSMEKHKFYLTMTQADKAALCSRAK
6. Ba 1a KRDIVSKTFRCPQDVVSAVKILKRKCANRSSKYNGWVSSSKVEKSLSKSRIVSINQVSMEKHKFYLTMTEADKAALCSRAK
900 910 920 930 940 950 960 966
Consensus DVGLDKTWVESNIKTVHEAQGISVDHVVLVRLKSTKCDLFKSEEYCLVALTRHKKTFEYLYNGDLGGDLISFCVT
Identity
1. LyV 1a DVGLDKTWVESNIKTVHEAQGISVDHVVLVRLKSTKCDLFKSEEYCLVALTRHKKTFEYLYNGDLGGDLISFCVT
2. MV 1a DVGLDKTWVESNIKTVHEAQGISVDHVVLVRLKSTKCDLFKSEEYCLVALTRHKKTFEYLYNGDLGGDLISFCVT
3. SV 1a DVGLDKTWVESNIKTVHEAQGISVDHVVLVRLKSTKCDLFKSEEYCLVALTRHKKTFEYLYNGDLGGDLISFCVT
4. TU 1a DVGLDKTWVESNIKTVHEAQGISVDHVVLVRLKSTKCDLFKSEEYCLVALTRHKKTFEYLYNGDLGGDLISFCVT
5. TV 1a DVGLEKTWVESNIKTVHEAQGISVDHVVLVRLKSTKCDLFKSEEYCLVALTRHKKTFEYLYNGDLGGDLISFCVT
6. Ba 1a DVGLDKTWVESNMETVHEAQGKAVDHVVLVRLKSTKCDLFKSEEYCLVALTRHKRTFEYLYNGDLGGDLISFYVT
